# (1*S*,3*S*,8*R*,10*R*,11*R*)-3,7,7,10-Tetra­methyl­tri­cyclo­[6.4.0.0^1,3^]dodecan-11-ol

**DOI:** 10.1107/S1600536813020576

**Published:** 2013-07-31

**Authors:** Ahmed Benharref, Jamal El karroumi, Lahcen El Ammari, Mohamed Saadi, Moha Berraho

**Affiliations:** a Laboratoire de Chimie Biomoléculaires, Substances Naturelles et Réactivité, URAC16, Faculté des Sciences, Semlalia, BP 2390 Bd My Abdellah, 40000 Marrakech, Morocco; b Laboratoire de Chimie du Solide Appliquée, Faculté des Sciences, Université MohammedV-Agdal, Avenue Ibn Battouta, BP 1014, Rabat, Morocco; c Laboratoire de Chimie des Substances Naturelles ‘Unité Associé au CNRST (URAC16)’, Faculté des Sciences Semlalia, BP 2390 Bd My Abdellah, 40000 Marrakech, Morocco

## Abstract

The title compound, C_16_H_28_O, was synthesized by three steps from β-himachalene (3,5,5,9-tetra­methyl-2,4a,5,6,7,8-hexa­hydro-1*H*-benzo­cyclo­heptene), which was isolated from the essential oil of the Atlas cedar (*Cedrus atlantica*). The mol­ecule is built up from fused six- and seven-membered rings and an appended three-membered ring. The six-membered ring has twist-boat conformation, whereas the seven-membered ring displays a chair conformation. In the crystal, mol­ecules are linked into chains propagating along the *a-*axis direction by O—H⋯O hydrogen bonds.

## Related literature

For the reactivity and biological properties of β-himachalene, see: Auhmani *et al.*(2002 ▶); El Jamili *et al.* (2002 ▶); Daoubi *et al.* (2004 ▶). For related structures, see: Ourhriss *et al.* (2013 ▶); Benharref *et al.* (2013 ▶). For puckering parameters, see: Cremer & Pople (1975 ▶).
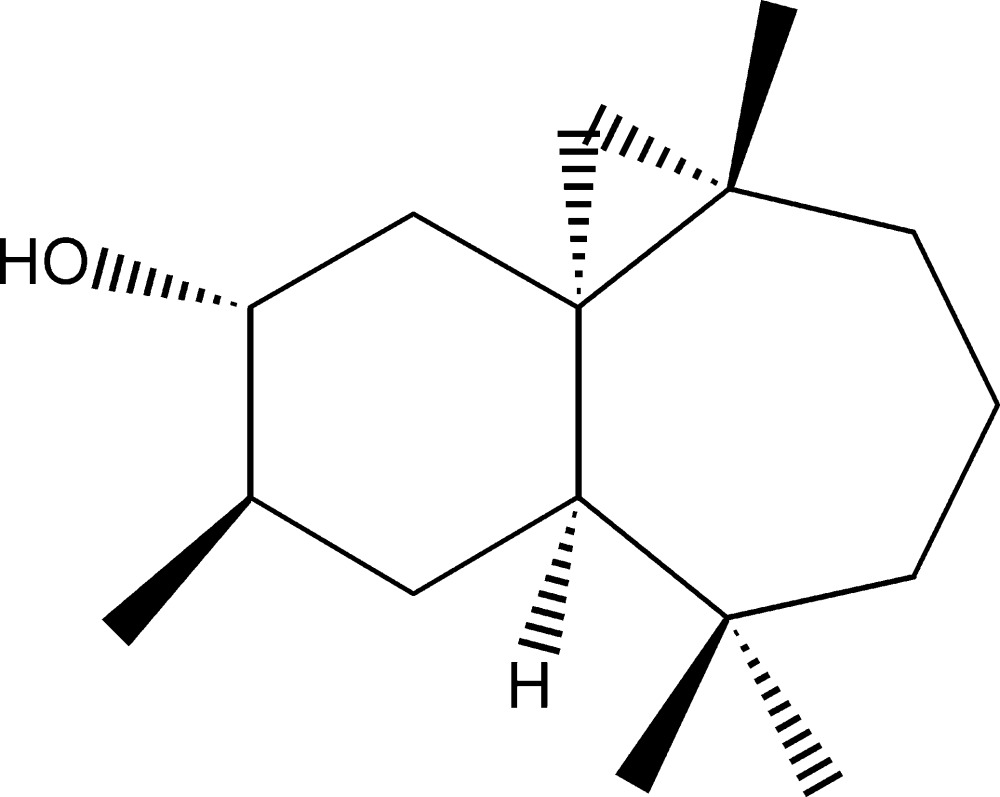


## Experimental

### 

#### Crystal data

C_16_H_28_O
*M* *_r_* = 236.38
Orthorhombic, 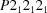

*a* = 5.8796 (2) Å
*b* = 12.7822 (4) Å
*c* = 19.1496 (7) Å
*V* = 1439.17 (8) Å^3^
*Z* = 4
Mo *K*α radiation
μ = 0.07 mm^−1^
*T* = 298 K
0.25 × 0.15 × 0.10 mm

#### Data collection

Bruker APEXII CCD diffractometer
8482 measured reflections
1724 independent reflections
1485 reflections with *I* > 2σ(*I*)
*R*_int_ = 0.030

#### Refinement

*R*[*F* ^2^ > 2σ(*F* ^2^)] = 0.038
*wR*(*F* ^2^) = 0.102
*S* = 1.03
1724 reflections
160 parameters
H-atom parameters constrained
Δρ_max_ = 0.15 e Å^−3^
Δρ_min_ = −0.12 e Å^−3^

### 

Data collection: *APEX2* (Bruker, 2009 ▶); cell refinement: *SAINT* (Bruker, 2009 ▶); data reduction: *SAINT*; program(s) used to solve structure: *SHELXS97* (Sheldrick, 2008 ▶); program(s) used to refine structure: *SHELXL97* (Sheldrick, 2008 ▶); molecular graphics: *ORTEP-3 for Windows* (Farrugia, 2012 ▶) and *PLATON* (Spek, 2009 ▶); software used to prepare material for publication: *WinGX* (Farrugia, 2012 ▶).

## Supplementary Material

Crystal structure: contains datablock(s) I. DOI: 10.1107/S1600536813020576/bt6923sup1.cif

Structure factors: contains datablock(s) I. DOI: 10.1107/S1600536813020576/bt6923Isup2.hkl

Supplementary material file. DOI: 10.1107/S1600536813020576/bt6923Isup3.cml

Additional supplementary materials:  crystallographic information; 3D view; checkCIF report

## Figures and Tables

**Table 1:** Hydrogen-bond geometry (Å, °)

*D*—H⋯*A*	*D*—H	H⋯*A*	*D*⋯*A*	*D*—H⋯*A*
O1—H1⋯O1^i^	0.82	2.35	3.139 (3)	163

Symmetry code: (i) 
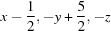
.

**Fig. 1.**
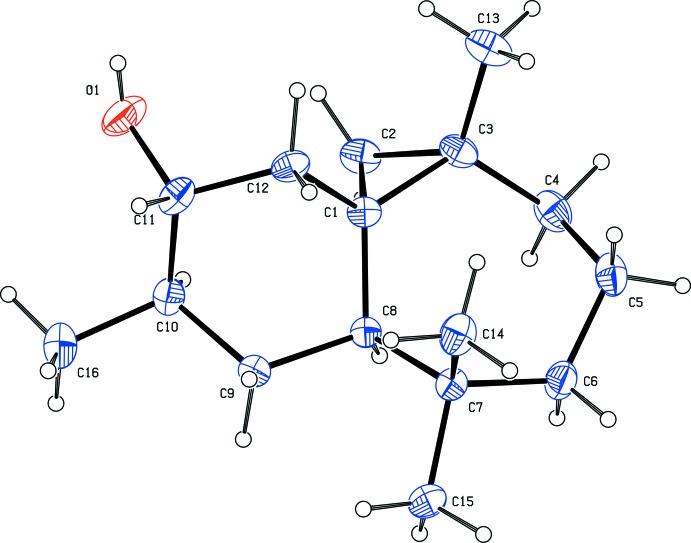
Molecular structure of the title compound with the atom-labelling scheme. Displacement ellipsoids are drawn at the 30% probability. level. H atoms are represented as small spheres of arbitrary radii.

**Fig. 2.**
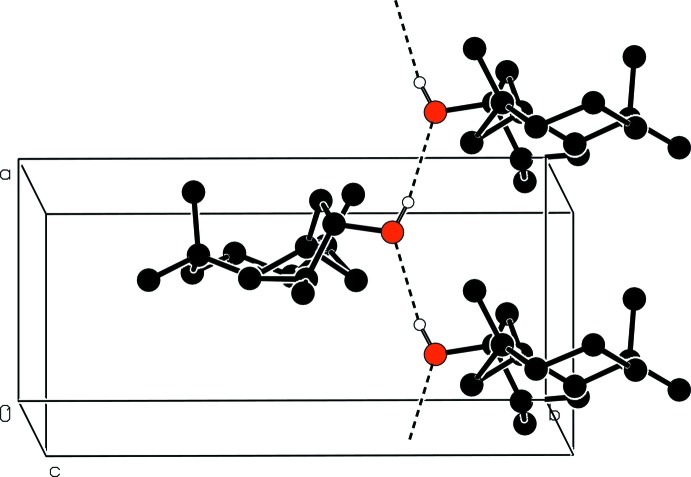
Partial packing view showing the O–H···O interactions (dashed lines)and the formation of a chain parallel to the a axis. H atoms not involved in hydrogen bonding have been omitted for clarity.

## supplementary crystallographic information

### Comment

The essential oil of the Atlas cedar (Cedrus atlantica) consists mainly (50%) of a bicyclic hydrocarbon sesquiterpene called β-himachalene (El Jamili *et al.*, 2002). The reactivity of this terpene and its derivatives has been studied extensively by our team in order to prepare new products having biological properties (Daoubi *et al.*, 2004; Benharref *et al.* 2013; Ourhriss *et al.*, 2013). In this work, We present the crystal structure of (1*S*, 3S,8*R*, 10*R*, 11*R*)-3,7,7,10-tetramethyltricyclo[6.4.0.0^1^,^3^] dodec-11-ol. The molecule is built up from two fused six- and seven-membered rings and an additional three-membered ring (Fig. 1). The six-membered ring has a twist-boat conformation, as indicated by the total puckering amplitude QT = 0.783 (2) Å and spherical polar angle θ = 90.98 (15)° with φ = 334.28 (16)°, whereas the seven-membered ring displays a chair conformation with QT = 0.7646 (22) Å, θ2 = 32.19 (19), φ2 = 128.95 (34) and φ3 = 101.54 (20)° (Cremer & Pople, 1975). In the crystal structure, molecules are linked into chains (Fig. 2) running along the *a* axis by intermolecular O–H···O hydrogen bonds (Table 1).

### Experimental

In a three-necked flask equipped with a dropping funnel, a condenser and a magnetic stirrer, maintained at 0°C, 2 g (6.6 mmol) of (1*S*,3*R*,8*R*) - 2,2-Dichloro-3,7,7,10-tetramethyltricyclo-[6.4.0.0^1^,^3^] dodec-9-en-11-one (Auhmani *et al.*, 2002) were introduced in 50 ml of ether. Then, 2 g of sodium were added by small portions during one hour, and dropwise 65 ml of a methanol solution 2.5% of water. The reaction mixture was stirred for 12 h. After hydrolysis with 20 mL of water, the two phases were separated and the aqueous phase was extracted three times with 20 ml of ether. The organic phases were combined and dried over sodium sulfate and concentrated. The residue obtained was chromatographed on a silica column with hexane and ethyl acetate(95/5) as eluent to give the sesquiterpene alcohol (1*S*, 3S,8*R*, 10*R*, 11*R*)-3,7,7,10- tetramethyltricyclo[6.4.0.0^1^,^3^]dodec-11-ol with a yield of 97% (1.5 g; 6.4 mmol). The title compound was recrystallized from n-pentane.

### Refinement

All H atoms were fixed geometrically and treated as riding with C—H = 0.96 Å (methyl), 0.97 Å (methylene), 0.98 Å (methine) and 0.82 Å (O–H) with *U*_iso_(H) = 1.2U_eq_ (methylene, methine) or *U*_iso_(H) = 1.5*U*_eq_ (methyl and OH). The methyl groups and the hydroxyl group were allowed to rotate but not to tip. In the absence of significant anomalous scattering, the absolute configuration could not be reliably determined and thus Friedel pairs were merged and any references to the Flack parameter were removed.

### Crystal data

**Table:**

C_16_H_28_O	*F*(000) = 528
*M**_r_* = 236.38	*D*_x_ = 1.091 Mg m^−^^3^
Orthorhombic, *P*2_1_2_1_2_1_	Mo *K*α radiation, λ = 0.71073 Å
Hall symbol: P 2ac 2ab	Cell parameters from 1724 reflections
*a* = 5.8796 (2) Å	θ = 2.7–26.4°
*b* = 12.7822 (4) Å	µ = 0.07 mm^−^^1^
*c* = 19.1496 (7) Å	*T* = 298 K
*V* = 1439.17 (8) Å^3^	Prism, colourless
*Z* = 4	0.25 × 0.15 × 0.10 mm

### Data collection

**Table:**

Bruker APEXII CCD diffractometer	1485 reflections with *I* > 2σ(*I*)
Radiation source: fine-focus sealed tube	*R*_int_ = 0.030
Graphite monochromator	θ_max_ = 26.4°, θ_min_ = 2.7°
ω and φ scans	*h* = −7→6
8482 measured reflections	*k* = −15→15
1724 independent reflections	*l* = −23→23

### Refinement

**Table:**

Refinement on *F*^2^	Secondary atom site location: difference Fourier map
Least-squares matrix: full	Hydrogen site location: inferred from neighbouring sites
*R*[*F*^2^ > 2σ(*F*^2^)] = 0.038	H-atom parameters constrained
*wR*(*F*^2^) = 0.102	*w* = 1/[σ^2^(*F*_o_^2^) + (0.0536*P*)^2^ + 0.1784*P*] where *P* = (*F*_o_^2^ + 2*F*_c_^2^)/3
*S* = 1.03	(Δ/σ)_max_ < 0.001
1724 reflections	Δρ_max_ = 0.15 e Å^−^^3^
160 parameters	Δρ_min_ = −0.12 e Å^−^^3^
0 restraints	Extinction correction: *SHELXL97* (Sheldrick, 2008), Fc^*^=kFc[1+0.001xFc^2^λ^3^/sin(2θ)]^-1/4^
Primary atom site location: structure-invariant direct methods	Extinction coefficient: 0.012 (3)

### Special details

**Table:**

Geometry. All s.u.'s (except the s.u. in the dihedral angle between two l.s. planes) are estimated using the full covariance matrix. The cell s.u.'s are taken into account individually in the estimation of s.u.'s in distances, angles and torsion angles; correlations between s.u.'s in cell parameters are only used when they are defined by crystal symmetry. An approximate (isotropic) treatment of cell s.u.'s is used for estimating s.u.'s involving l.s. planes.

### Fractional atomic coordinates and isotropic or equivalent isotropic displacement parameters (Å^2^)

**Table:**

	*x*	*y*	*z*	*U*_iso_*/*U*_eq_	
C1	0.8389 (3)	1.04007 (14)	0.11966 (10)	0.0381 (4)	
C2	0.9711 (4)	1.13257 (15)	0.14824 (11)	0.0510 (5)	
H2A	1.1339	1.1244	0.1544	0.061*	
H2B	0.9231	1.2021	0.1342	0.061*	
C3	0.8171 (4)	1.07206 (16)	0.19602 (11)	0.0467 (5)	
C4	0.9300 (4)	1.00520 (18)	0.25137 (11)	0.0585 (6)	
H4A	0.9184	1.0411	0.2958	0.070*	
H4B	1.0904	0.9991	0.2402	0.070*	
C5	0.8329 (5)	0.89612 (19)	0.25971 (11)	0.0612 (6)	
H5A	0.6687	0.8995	0.2553	0.073*	
H5B	0.8678	0.8707	0.3062	0.073*	
C6	0.9253 (4)	0.81891 (16)	0.20627 (10)	0.0551 (6)	
H6A	0.8718	0.7499	0.2194	0.066*	
H6B	1.0896	0.8183	0.2108	0.066*	
C7	0.8694 (3)	0.83462 (15)	0.12864 (10)	0.0413 (5)	
C8	0.9669 (3)	0.93978 (13)	0.09937 (9)	0.0352 (4)	
H8	1.1210	0.9467	0.1183	0.042*	
C9	0.9916 (4)	0.93761 (14)	0.01895 (9)	0.0439 (4)	
H9A	1.1288	0.8996	0.0068	0.053*	
H9B	0.8634	0.9003	−0.0009	0.053*	
C10	1.0027 (4)	1.04668 (16)	−0.01305 (10)	0.0489 (5)	
H10	1.1151	1.0873	0.0133	0.059*	
C11	0.7729 (4)	1.09873 (15)	−0.00364 (12)	0.0528 (6)	
H11	0.6729	1.0760	−0.0417	0.063*	
C12	0.6602 (4)	1.06999 (15)	0.06595 (11)	0.0464 (5)	
H12A	0.5728	1.1291	0.0830	0.056*	
H12B	0.5567	1.0118	0.0590	0.056*	
C13	0.5996 (5)	1.1233 (2)	0.22183 (14)	0.0690 (7)	
H13A	0.6296	1.1599	0.2646	0.104*	
H13B	0.4863	1.0706	0.2298	0.104*	
H13C	0.5456	1.1719	0.1874	0.104*	
C14	0.6117 (4)	0.82570 (18)	0.11891 (13)	0.0562 (6)	
H14A	0.5752	0.8317	0.0702	0.084*	
H14B	0.5375	0.8807	0.1443	0.084*	
H14C	0.5607	0.7591	0.1361	0.084*	
C15	0.9811 (5)	0.74201 (14)	0.09077 (12)	0.0563 (6)	
H15A	0.9325	0.6777	0.1119	0.084*	
H15B	1.1435	0.7478	0.0942	0.084*	
H15C	0.9371	0.7426	0.0425	0.084*	
C16	1.0780 (6)	1.0442 (2)	−0.08917 (12)	0.0769 (8)	
H16A	1.2321	1.0197	−0.0919	0.115*	
H16B	1.0686	1.1134	−0.1085	0.115*	
H16C	0.9807	0.9980	−0.1150	0.115*	
O1	0.8089 (4)	1.20972 (12)	−0.01013 (12)	0.0789 (6)	
H1	0.6857	1.2396	−0.0120	0.118*	

### Atomic displacement parameters (Å^2^)

**Table:**

	*U*^11^	*U*^22^	*U*^33^	*U*^12^	*U*^13^	*U*^23^
C1	0.0310 (9)	0.0379 (9)	0.0454 (10)	−0.0016 (8)	−0.0027 (9)	−0.0033 (8)
C2	0.0448 (12)	0.0457 (10)	0.0625 (13)	−0.0076 (10)	−0.0022 (11)	−0.0117 (9)
C3	0.0404 (11)	0.0495 (11)	0.0502 (11)	−0.0040 (10)	0.0018 (10)	−0.0143 (9)
C4	0.0555 (14)	0.0780 (15)	0.0419 (11)	−0.0040 (12)	−0.0058 (11)	−0.0128 (10)
C5	0.0680 (15)	0.0762 (15)	0.0394 (11)	−0.0016 (14)	0.0003 (12)	0.0067 (10)
C6	0.0609 (14)	0.0566 (12)	0.0478 (11)	0.0041 (11)	−0.0034 (11)	0.0126 (10)
C7	0.0406 (11)	0.0402 (9)	0.0431 (10)	−0.0007 (9)	−0.0014 (9)	0.0032 (8)
C8	0.0285 (9)	0.0394 (9)	0.0378 (9)	−0.0005 (8)	−0.0029 (8)	0.0002 (7)
C9	0.0476 (11)	0.0420 (9)	0.0419 (10)	0.0026 (9)	0.0028 (9)	−0.0010 (8)
C10	0.0540 (12)	0.0490 (11)	0.0435 (10)	−0.0043 (11)	−0.0007 (10)	0.0056 (9)
C11	0.0549 (13)	0.0448 (11)	0.0586 (12)	−0.0005 (10)	−0.0141 (11)	0.0099 (10)
C12	0.0375 (10)	0.0416 (10)	0.0603 (12)	0.0045 (9)	−0.0077 (10)	0.0004 (9)
C13	0.0592 (15)	0.0730 (16)	0.0748 (16)	0.0046 (14)	0.0130 (13)	−0.0234 (13)
C14	0.0478 (12)	0.0572 (13)	0.0635 (13)	−0.0135 (11)	−0.0021 (11)	0.0105 (11)
C15	0.0687 (16)	0.0371 (10)	0.0632 (13)	0.0014 (11)	0.0020 (13)	0.0016 (9)
C16	0.100 (2)	0.0785 (16)	0.0527 (13)	0.0024 (17)	0.0111 (15)	0.0157 (12)
O1	0.0872 (13)	0.0459 (8)	0.1035 (14)	0.0081 (9)	0.0012 (13)	0.0243 (9)

### Geometric parameters (Å, º)

**Table:**

C1—C2	1.517 (3)	C9—H9A	0.9700
C1—C12	1.519 (3)	C9—H9B	0.9700
C1—C3	1.524 (3)	C10—C11	1.517 (3)
C1—C8	1.536 (2)	C10—C16	1.524 (3)
C2—C3	1.502 (3)	C10—H10	0.9800
C2—H2A	0.9700	C11—O1	1.440 (2)
C2—H2B	0.9700	C11—C12	1.533 (3)
C3—C4	1.515 (3)	C11—H11	0.9800
C3—C13	1.520 (3)	C12—H12A	0.9700
C4—C5	1.515 (3)	C12—H12B	0.9700
C4—H4A	0.9700	C13—H13A	0.9600
C4—H4B	0.9700	C13—H13B	0.9600
C5—C6	1.522 (3)	C13—H13C	0.9600
C5—H5A	0.9700	C14—H14A	0.9600
C5—H5B	0.9700	C14—H14B	0.9600
C6—C7	1.536 (3)	C14—H14C	0.9600
C6—H6A	0.9700	C15—H15A	0.9600
C6—H6B	0.9700	C15—H15B	0.9600
C7—C14	1.531 (3)	C15—H15C	0.9600
C7—C15	1.536 (3)	C16—H16A	0.9600
C7—C8	1.565 (2)	C16—H16B	0.9600
C8—C9	1.547 (2)	C16—H16C	0.9600
C8—H8	0.9800	O1—H1	0.8200
C9—C10	1.524 (3)		
			
C2—C1—C12	113.73 (16)	C10—C9—H9A	109.0
C2—C1—C3	59.18 (13)	C8—C9—H9A	109.0
C12—C1—C3	121.59 (17)	C10—C9—H9B	109.0
C2—C1—C8	119.38 (16)	C8—C9—H9B	109.0
C12—C1—C8	112.19 (15)	H9A—C9—H9B	107.8
C3—C1—C8	120.51 (16)	C11—C10—C16	112.4 (2)
C3—C2—C1	60.63 (13)	C11—C10—C9	108.36 (18)
C3—C2—H2A	117.7	C16—C10—C9	112.22 (18)
C1—C2—H2A	117.7	C11—C10—H10	107.9
C3—C2—H2B	117.7	C16—C10—H10	107.9
C1—C2—H2B	117.7	C9—C10—H10	107.9
H2A—C2—H2B	114.8	O1—C11—C10	106.89 (19)
C2—C3—C4	116.9 (2)	O1—C11—C12	112.0 (2)
C2—C3—C13	118.91 (19)	C10—C11—C12	112.53 (17)
C4—C3—C13	112.6 (2)	O1—C11—H11	108.4
C2—C3—C1	60.18 (12)	C10—C11—H11	108.4
C4—C3—C1	118.90 (17)	C12—C11—H11	108.4
C13—C3—C1	119.91 (19)	C1—C12—C11	110.48 (17)
C3—C4—C5	115.3 (2)	C1—C12—H12A	109.6
C3—C4—H4A	108.4	C11—C12—H12A	109.6
C5—C4—H4A	108.4	C1—C12—H12B	109.6
C3—C4—H4B	108.4	C11—C12—H12B	109.6
C5—C4—H4B	108.4	H12A—C12—H12B	108.1
H4A—C4—H4B	107.5	C3—C13—H13A	109.5
C4—C5—C6	113.0 (2)	C3—C13—H13B	109.5
C4—C5—H5A	109.0	H13A—C13—H13B	109.5
C6—C5—H5A	109.0	C3—C13—H13C	109.5
C4—C5—H5B	109.0	H13A—C13—H13C	109.5
C6—C5—H5B	109.0	H13B—C13—H13C	109.5
H5A—C5—H5B	107.8	C7—C14—H14A	109.5
C5—C6—C7	119.33 (18)	C7—C14—H14B	109.5
C5—C6—H6A	107.5	H14A—C14—H14B	109.5
C7—C6—H6A	107.5	C7—C14—H14C	109.5
C5—C6—H6B	107.5	H14A—C14—H14C	109.5
C7—C6—H6B	107.5	H14B—C14—H14C	109.5
H6A—C6—H6B	107.0	C7—C15—H15A	109.5
C14—C7—C6	108.64 (19)	C7—C15—H15B	109.5
C14—C7—C15	108.0 (2)	H15A—C15—H15B	109.5
C6—C7—C15	105.37 (16)	C7—C15—H15C	109.5
C14—C7—C8	112.51 (17)	H15A—C15—H15C	109.5
C6—C7—C8	112.38 (16)	H15B—C15—H15C	109.5
C15—C7—C8	109.65 (16)	C10—C16—H16A	109.5
C1—C8—C9	108.22 (15)	C10—C16—H16B	109.5
C1—C8—C7	116.53 (15)	H16A—C16—H16B	109.5
C9—C8—C7	112.05 (14)	C10—C16—H16C	109.5
C1—C8—H8	106.5	H16A—C16—H16C	109.5
C9—C8—H8	106.5	H16B—C16—H16C	109.5
C7—C8—H8	106.5	C11—O1—H1	109.5
C10—C9—C8	112.81 (15)		

### Hydrogen-bond geometry (Å, º)

**Table:**

*D*—H···*A*	*D*—H	H···*A*	*D*···*A*	*D*—H···*A*
O1—H1···O1^i^	0.82	2.35	3.139 (3)	163

Symmetry code: (i) *x*−1/2, −*y*+5/2, −*z*.
